# Collaborative representation and confidence-driven semi-supervised learning for hyperspectral image classification

**DOI:** 10.1038/s41598-026-36806-6

**Published:** 2026-01-24

**Authors:** Yutian Chen, Hongliang Lu, Xianglin Huang

**Affiliations:** 1 School of Geography and Planning, Huaiyin Normal University, Huai’an, 223300 China; 2 School of Architectural Engineering, Tongling University, Tongling, 244061 China; 3 Anhui Province Joint Construction Discipline Key Laboratory of Spatial Information Acquisition and Application, Tongling University, Tongling, 244061 China; 4 School of Earth Science and Engineering, Hohai University, Nanjing, 211100 China

**Keywords:** Dynamic ensemble learning, Hyperspectral image classification, Graph-convolutional networks, Engineering, Mathematics and computing

## Abstract

Hyperspectral image (HSI) classification faces challenges in diverse scenarios due to spectral-spatial complexity and class imbalance. Existing methods lack generalizability. This paper presents a novel Graph-Convolutional Networks with Adaptive Region Ensembles (GCN-ARE) framework. It integrates graph spectral learning, dynamic region subdivision, and classifier fusion. The key contributions are as follows: First, a normalized graph Laplacian operator ensures graph spectral stability, bounding the eigenvalue spectrum to stabilize feature propagation and address gradient issues in irregular terrains. Second, recursive K-means clustering under empirical risk bounds achieves adaptive region optimality, dynamically partitioning complex regions for enhanced local discriminability. Third, theoretical guarantees based on Hoeffding’s inequality enable dynamic ensemble consistency, facilitating optimal classifier selection under spatial-spectral uncertainty. Experiments on four HSI datasets (Botswana, Houston, Indian Pines, WHU-Hi-LongKou) show that GCN-ARE outperforms benchmarks like ViT and GAT, with average OA improvements of 1.5–5.7%. Ablation studies confirm the importance of adaptive subdivision and ensemble modules, and parameter sensitivity analyses reveal its robustness. The framework sets a new standard for robust HSI classification with its theoretical rigor and practical efficacy.

## Introduction

 Hyperspectral image (HSI) classification technology plays a crucial role in numerous fields such as environmental monitoring and precision agriculture, thanks to its ability to jointly process spectral and spatial information^1–3^. Through hyperspectral images, researchers can achieve accurate identification of land cover types, providing solid data support and evidence for various decision-making processes. However, the inherent high dimensionality, spectral similarity between classes, and severe class imbalance in hyperspectral images pose significant challenges to the generalization ability of classification models^4,5^. Traditional machine learning methods rely too heavily on manually designed features and struggle to fully explore the complex patterns within the data. Deep learning models^6–10^, such as convolutional neural network (CNN) and Transformer, can automatically extract features but are limited by local receptive fields.

**Fig. 1:**
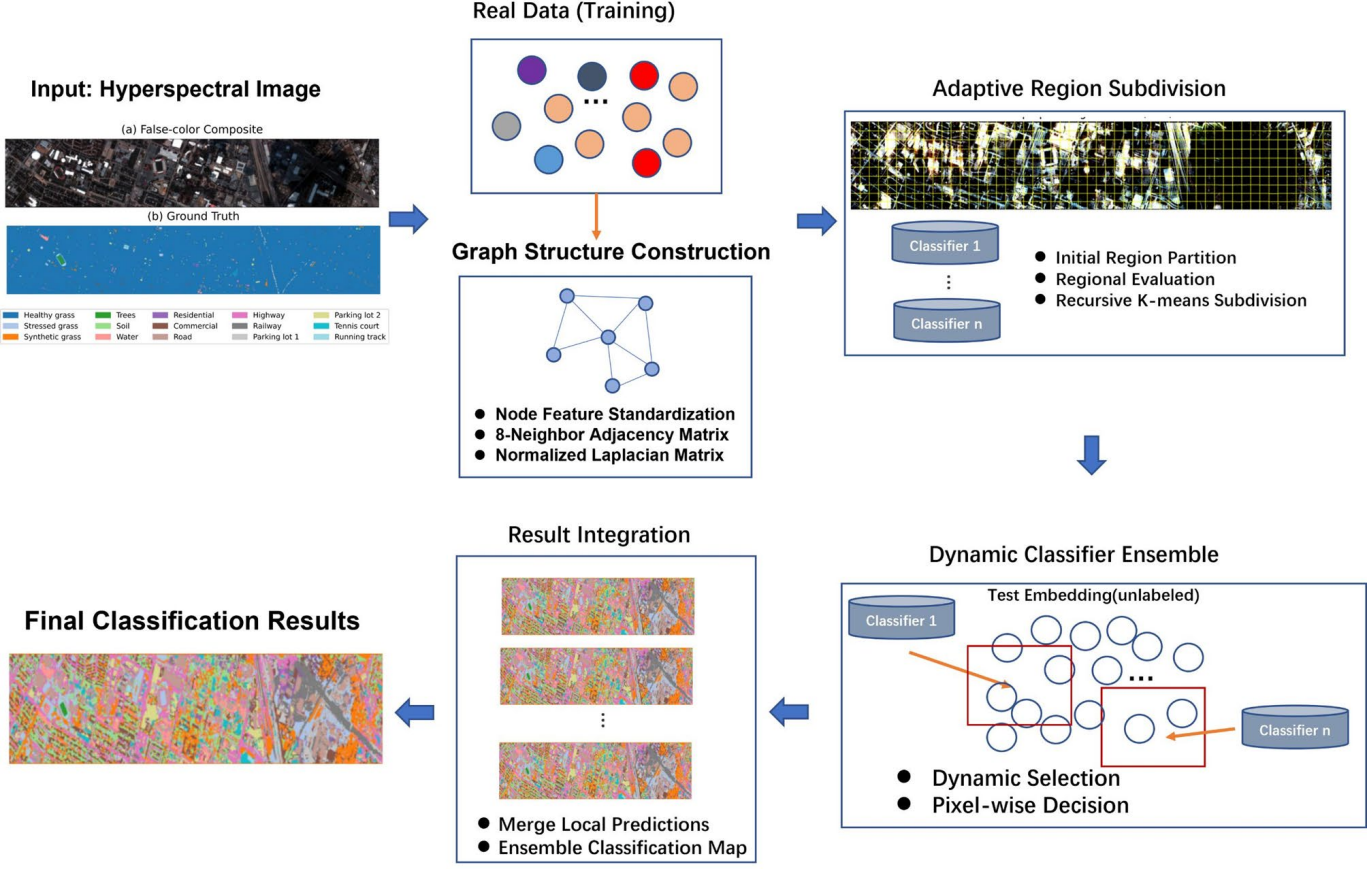
Flow diagram of the proposed GCN-ARE framework. The GCN-ARE framework comprises four key modules: (1) Graph construction with normalized Laplacian, (2) Supervised GCN training, (3) Recursive region subdivision via empirical risk bounds, and (4) Dynamic classifier ensemble with Hoeffding’s inequality and high computational complexity, respectively. Therefore, constructing a classification framework that can effectively model spectral-spatial relationships and flexibly adapt to heterogeneous terrains has become a core issue to be urgently addressed in the field of hyperspectral image analysis.

Currently, HSI classification methods can be mainly categorized into the following two types: The first type is traditional machine learning methods, which rely on manually designed features and have significant limitations in mining complex features of data^11,12^. Support vector machines (SVM) and random forests are typical representatives, usually performing classification tasks with the help of hand-crafted texture features, spectral indices, etc^13^. Although these methods are computationally efficient, they often fall short when dealing with complex nonlinear relationships^14^. In recent years, some studies have attempted to improve traditional methods. For example, reference^15^ proposed a method that combines local binary patterns (LBP) with SVM, enhancing the utilization of texture information and achieving certain results on some datasets. However, in complex scenarios, the inherent limitations of hand-crafted features are still obvious^16^. The second type is deep learning methods^17–22^. Deep learning models represented by CNNs and Transformers have the ability to automatically extract features but still suffer from issues such as local receptive fields or high computational complexity^23,24^. 3D-CNNs and vision Transformers are important research directions in this field. CNNs obtain local spatial features through convolutional layers but perform poorly when dealing with irregular terrains such as fragmented vegetation areas in the Botswana dataset. Although ViT can capture global dependencies, due to its quadratic computational complexity, it faces scalability challenges in large-scale data processing^25,26^. In addition, due to heuristic design and the inconsistency between feature extraction and classification objectives, it is difficult to optimize the model’s generalization performance.

In addition to the above problems, existing HSI classification methods also face difficulties in classifier selection. In practical application scenarios, there are various types of classifiers, and their performances vary significantly across different datasets and scenarios. When selecting classifiers, traditional machine learning methods often lack scientific and systematic bases and mostly rely on empirical judgment, making it difficult to achieve the best classification results. For example, when dealing with hyperspectral images with complex texture and spectral features, different combinations of hand-crafted features with various classifiers may produce vastly different results, but traditional methods lack effective means to determine the optimal combination. Although deep learning methods have the advantage of automatic feature extraction, they also have deficiencies in the classifier selection stage. Due to the lack of a quantitative evaluation mechanism for the performance of different classifiers in specific areas, it is difficult to.

**Fig. 2:**
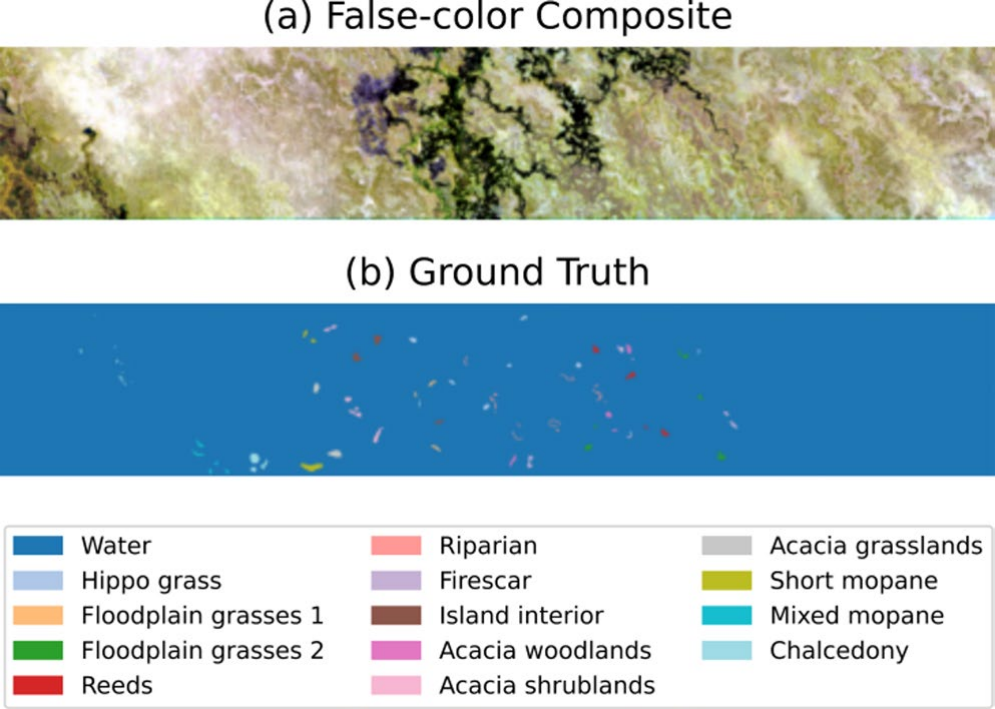
(**a**) False-color image and (**b**) ground truth of Botswana dataset.

**Table 1:** Sample description in Botswana dataset.

No.	class	samples
*1*	Water	270
*2*	Hippo grass	101
*3*	Floodplain grasses 1	251
*4*	Floodplain grasses 2	215
*5*	Reeds	269
*6*	Riparian	269
*7*	Firescar	259
*8*	Island interior	203
*9*	Acacia woodlands	314
*10*	Acacia shrublands	248
*11*	Acacia grasslands	305
*12*	Short mopane	181
*13*	Mixed mopane	268
*14*	Chalcedony	95
	Total	3248

accurately determine which classifier can process regional data more efficiently when dealing with spatial-spectral uncertainties. For example, in complex terrain areas, different CNN architectures or Transformer variants have their own advantages and disadvantages in feature extraction and classification decision-making. However, existing deep learning methods lack theoretical support, making it difficult to precisely select the optimal classifier, thus limiting the model’s generalization performance and its adaptability to diverse practical application scenarios. This blindness in classifier selection not only reduces classification accuracy but also causes the model to perform unstable when facing new data or different scenarios, severely restricting the potential of HSI classification technology.

**Fig. 3:**
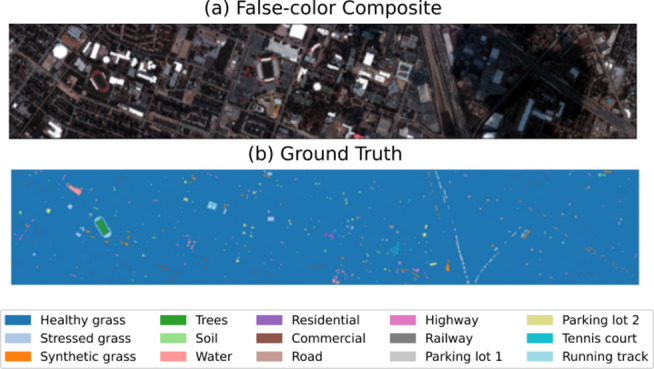
(**a**) False-color image and (**b**) ground truth of Houston dataset.

**Table 2:** Sample description in Houston dataset.

No.	class	samples
*1*	Healthy grass	1251
*2*	Stressed grass	1254
*3*	Synthetic grass	697
*4*	Trees	1244
*5*	Soil	1242
*6*	Water	325
*7*	Residential	1268
*8*	Commercial	1244
*9*	Road	1252
*10*	Highway	1227
*11*	Railway	1235
*12*	Parking lot1	1233
*13*	Parking lot2	469
*14*	Tennis court	428
*15*	Running track	660
	Total	15,029

To address the above problems, this paper proposes a graph-convolutional networks with adaptive region ensembles (GCN-ARE), aiming to effectively overcome the deficiencies of existing methods. The main innovations are as follows:

1. Graph spectrum stability guarantee: By utilizing the normalized graph Laplacian operator, the eigenvalue spectrum is ensured to be bounded, effectively alleviating the problems of gradient explosion and vanishing, and enhancing the stability of feature learning in complex scenarios.
2. Dynamic region optimization: Recursive K-means clustering based on the empirical risk bound is employed to automatically identify complex regions, dynamically adjust the division, enhance the discriminability of local features, and improve the classification accuracy.

**Fig. 4:**
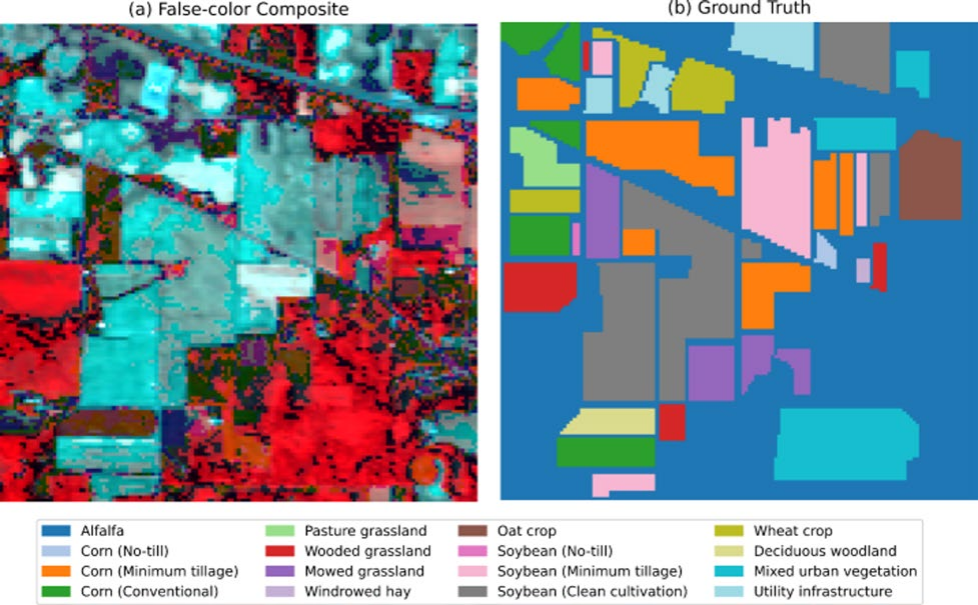
(**a**) False-color image and **(b**) ground truth of Indian Pines dataset.

**Table 3:** Sample description in Indian Pines dataset.

No.	class	samples
*1*	Alfalfa	46
*2*	Corn-notill	1428
*3*	Corn-min	830
*4*	Corn	237
*5*	Grass/Pasture	483
*6*	Grass/Trees	730
*7*	Grass/pasture-mowed	28
*8*	Hay-windrowed	478
*9*	Oats	20
*10*	Soybeans-notill	972
*11*	Soybeans-min	2455
*12*	Soybeans-clean	593
*13*	Wheat	205
*14*	Woods	1265
*15*	Bldg-Grass-Tree-Drives	386
*16*	Stone-steel towers	93
	Total	10,249

3. Classifier selection optimization: The Hoeffding’s inequality is leveraged to quantitatively analyze the performance of classifiers. The optimal classifier is selected in uncertain regions to ensure the reliability of decision-making.

The subsequent structure of this paper is arranged as follows: Section II elaborates on the GCN-ARE framework in detail; Section III validates the model performance through experiments; Section IV summarizes the research achievements and discusses future research directions.

**Fig. 5:**
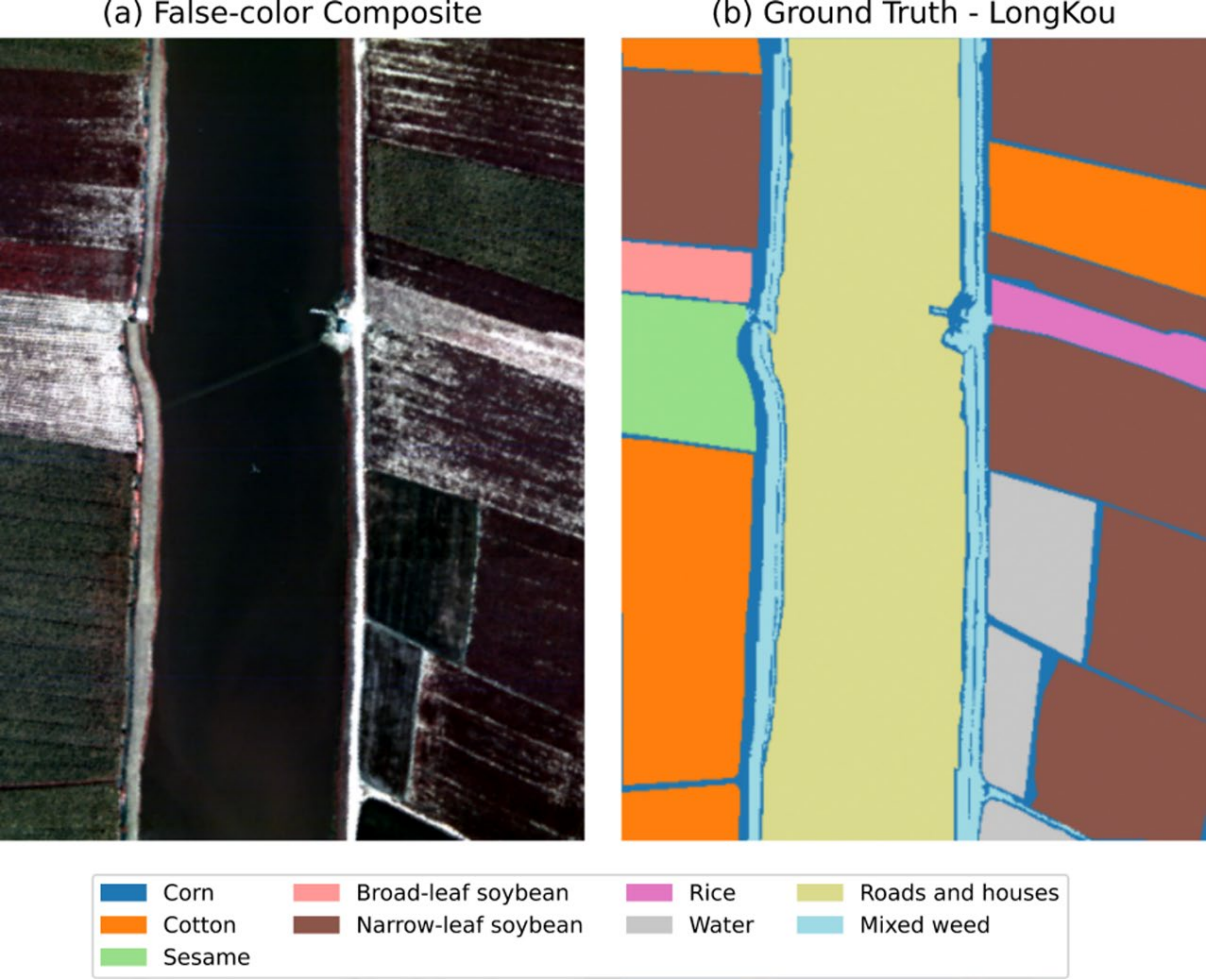
(**a**) False-color image and (**b**) ground truth of WHU-Hi-LongKou dataset.

**Table 4:** Sample description in WHU-Hi-LongKou dataset.

No.	class	samples
*1*	Corn	34,511
*2*	Cotton	8374
*3*	Sesame	3031
*4*	Broad-leaf soybean	63,212
*5*	Narrow-leaf soybean	4151
*6*	Rice	11,854
*7*	Water	67,056
*8*	Roads and houses	7124
*9*	Mixed weed	5229
	Total	204,542

## Methods

Despite the proliferation of HSI classification algorithms (e.g., SVM, CNN, Transformer), most methods exhibit inconsistent performance across diverse scenarios, primarily due to noisy graph construction and unstable propagation, strong spatial heterogeneity in land-cover distributions, and region-dependent classifier reliability. To address these challenges, we introduce three key design principles with theoretical guarantees:

1. **Graph Spectral Stability.** Explicit modeling of spectral-spatial relationships via normalized graph Laplacian operators, ensuring stable feature propagation.
2. **Adaptive Region Optimality.** Provable guarantees on region subdivision via recursive K-means clustering under empirical risk bounds.
3. **Dynamic Ensemble Consistency.** Theoretical analysis of classifier selection consistency under spatial-spectral uncertainty.

Existing methods often excel in specific scenarios but lack generalizability. By integrating graph-convolutional learning with adaptive region ensembles, our framework dynamically combines complementary classifiers, achieving robustness against spectral-spatial complexity. This section details our methodology for HSI classification, which integrates graph-convolutional feature learning with adaptive region subdivision and classifier ensembles. The flowchart of proposed method is presented in Fig. 1.

### Graph structure construction

A core challenge in HSI classification lies in modeling complex spatial-spectral relationships. Traditional CNNs, constrained by fixed receptive fields, struggle to represent irregular terrains and fragmented land-cover distributions. This module proposes a graph-based approach to explicitly encode spatial adjacency and spectral dependencies between pixels. Spatial edges are defined via 8-neighborhood connectivity.

1. **Node Features**: For pixel $\:i$ with spectral vector $\:{\mathbf{b}}_{i}\in\:{\mathbb{R}}^{C}$, normalized features are computed as

1 $$\:{\mathbf{x}}_{i}=\frac{{\mathbf{b}}_{i}-{\mu\:}_{\mathcal{B}}}{{\sigma\:}_{\mathcal{B}}}$$

where $\:{\mu\:}_{\mathcal{B}}$​ and $\:{\sigma\:}_{\mathcal{B}}$​ are the mean and standard deviation of valid pixel.

2. **Adjacency Matrix**: Define spatial connectivity via 8-neighborhood

2 $$\:{\mathbf{A}}_{ij}=\left\{\begin{array}{c}1\:\:\:\:\mathrm{i}\mathrm{f}\text{}\mathrm{max}\left(\left|{h}_{i}-{h}_{j}\right|,\left|{w}_{i}-{w}_{j}\right|\right)\le\:1\text{}\mathrm{a}\mathrm{n}\mathrm{d}\text{}i,j\in\:M\\\:0\:\:\:\:otherwise\:\:\:\:\:\:\:\:\:\:\:\:\:\:\:\:\:\:\:\:\:\:\:\:\:\:\:\:\:\:\:\:\:\:\:\:\:\:\:\:\:\:\:\:\:\:\:\:\:\:\:\:\:\:\:\:\:\:\:\:\:\:\:\:\:\end{array}\right.$$

where $\:\mathcal{M}$ is the set of valid pixels, $\:{h}_{i}$, $\:{w}_{i}$ note row and column indices of pixel $\:i$.Here, the distance between two pixels refers exclusively to their spatial-grid distance on the image lattice. Specifically, we adopt the Chebyshev distance in the 2D image coordinate system, where two pixels are considered spatial neighbors if the maximum difference in their row and column indices does not exceed one. No spectral distance is involved in Eq. (2); spectral information is encoded solely in the node features.

3. **Normalized Laplacian**: To stabilize training, the adjacency matrix is normalized as

3 $$\:\widehat{\mathbf{A}}={\stackrel{\sim}{\mathbf{D}}}^{-1/2}\left(\mathbf{A}+\mathbf{I}\right){\stackrel{\sim}{\mathbf{D}}}^{-1/2}$$

where $\:\stackrel{\sim}{\mathbf{D}}$ is the degree matrix with

4 $$\:{\stackrel{\sim}{\mathbf{D}}}_{ii}=1+{\sum\:}_{j}{\mathbf{A}}_{ij}$$

### Supervised GCN training

Extracting discriminative spectral-spatial features under limited labeled data remains a critical challenge for HSI classification. This module designs a two-layer graph-convolutional network (GCN) that integrates local and global contextual information through hierarchical feature propagation.

1. **GCN Propagation**: For input features $\:\mathbf{X}\in\:{\mathbb{R}}^{N\times\:C}$, the *l*-th layer output is

5 $$\:{\mathbf{H}}^{\left(l+1\right)}=\mathrm{ReLU}\left(\widehat{\mathbf{A}}{\mathbf{H}}^{\left(l\right)}{\mathbf{W}}^{\left(l\right)}\right)$$

where $\:{\mathbf{W}}^{\left(l\right)}$ is the learnable weight matrix of layer *l*.

#### Theorem 1

**(Graph Spectral Stability).** Let $\:\widehat{\mathbf{A}}$ be the normalized adjacency matrix (Eq. 3) and $\:\mathbf{L}=\mathbf{I}-\widehat{\mathbf{A}}$ the graph Laplacian. For any input feature $\:\mathbf{X}$, the eigenvalue spectrum $\:{\lambda\:}_{i}$ ​ of $\:\mathbf{L}$ satisfies

8 $$\:0\le\:{\lambda\:}_{i}\le\:2,\forall\:i\in\:1,\dots\:,N$$

**Table 5:** Configuration of the classifier pool used in the region-wise dynamic ensemble (DES).

Component	Base learner	Key settings (as used in our implementation)	Role in GCN-ARE
Lightweight classifier pool	Decision Tree (DT)	DecisionTreeClassifier (random_state = 42)	Candidate for region-wise selection
Lightweight classifier pool	SVM (RBF)	SVC (kernel=’rbf’, C = 1.0, gamma=’scale’, probability = True, random_state = 42)	Candidate for region-wise selection
Lightweight classifier pool	Random Forest (RF)	RandomForestClassifier (n_estimators = 100, random_state = 42)	Candidate for region-wise selection

**Table 6:** Summary of baseline configurations used in this paper.

Baseline	Implementation (library/module)	Key architecture hyperparameters	Training hyperparameters
**GCN**	PyTorch Geometric GCNConv	hidden dim = **256**; #GCN layers = **3**; ReLU after each layer; linear classifier Linear (256 → C)	Adam lr = **0.001**; epochs = **1000**; CE loss
**GAT**	PyTorch Geometric GATConv	hidden dim = **256**; heads = **4**; #GAT layers = **3**; (intermediate dim = 256×heads); ReLU; linear classifier Linear (256×heads → C)	Adam lr = **0.001**; epochs = **1000**; CE loss
**ViT**	PyTorch TransformerEncoder/Layer	token proj: Linear (D → 256); learnable pos embed (1 × 256); encoder layers = **3**; heads = **4**; FFN dim = **4 × 256**; mean pooling; classifier Linear (256 → C)	Adam lr = **0.001**; epochs = **1000**; CE loss
**Hybrid**	PyTorch (Conv1d + Transformer)	1D-CNN: Conv1d (1→64, k = 3) + MP (2) + Conv1d (64→128, k = 3) + MP (2); Transformer dim = **128**, heads = **2**, layers = **3**; classifier Linear (128 → C)	Adam lr = **0.001**; epochs = **1000**; CE loss
**EfficientFormer**	PyTorch (Conv1d embed + Transformer)	patch embed: Conv1d (1→64, k = 3) + GELU + Conv1d (64→128, k = 3, stride = 2) + GroupNorm; Transformer dim = **128**, heads = **4**, layers = **4**, FFN dim = **4 × 128**, GELU; mean pooling; classifier Linear (128 → C)	Adam lr = **0.001**; epochs = **800**; CE loss

#### Proof

The symmetric normalization in Eq. 3 ensures $\:\widehat{\mathbf{A}}$ is positive semi-definite with eigenvalues in [0,2]. Consequently, graph convolutions (Eq. 5) avoid gradient explosion/vanishing, stabilizing GCN training. This theorem rigorously justifies our graph construction—unlike fixed-grid CNNs, our spectral normalization adaptively controls feature smoothness across irregular terrains.

2. **Classification Layer**: Final predictions derive from

9 $$\:\widehat{\mathbf{Y}}=\mathrm{softmax}\left({\mathbf{H}}^{\left(2\right)}{\mathbf{W}}^{\left(2\right)}\right),\hspace{1em}{\mathbf{W}}^{\left(2\right)}\in\:{\mathbb{R}}^{d\times\:K}$$

where $\:K$ is the number of classes.

3. **Loss Function with Class Balancing**

10 $$\:\mathcal{L}=-{\sum\:}_{i\in\:{\mathcal{S}}_{\mathcal{k}}}\mathrm{log}{\widehat{y}}_{i}{y}_{i}$$

where $\:{n}_{k}={|\mathcal{S}}_{\mathcal{k}}|$ ensures equal influence across classes. The loss function (Eq. 10) introduces implicit class balancing. We formalize this through.

The heterogeneity of land-cover distributions in HSIs often leads to performance degradation in mixed regions (e.g., urban-rural transitions). This module proposes a recursive region subdivision strategy to dynamically identify and refine complex areas.

1. **Initial Partitioning**: Regions $\:{\mathcal{R}}_{\mathcal{k}}$ are defined by

11 $$\:{\mathcal{R}}_{\mathcal{k}}=\{\mathrm{i}\mid\:\mathrm{arg}\mathrm{max}\left({\widehat{\mathbf{Y}}}_{i}\right)=\mathcal{k}\}$$

2. **Region Evaluation**: For each $\:{\mathcal{R}}_{\mathcal{k}}$​, compute mean validation accuracy

12 $$\:{\stackrel{-}{\alpha\:}}_{\mathcal{k}}=\mathrm{Accuracy}\left({\mathcal{C}}_{\mathcal{m}},{\mathcal{D}}_{\mathrm{val}}^{\left(\mathcal{k}\right)}\right)$$

where $\:{\mathcal{C}}_{\mathcal{m}}$​ is the classifier, $\:{\mathcal{D}}_{\mathrm{val}}^{\left(\mathcal{k}\right)}$​ is the validation set of regions $\:k$.

3. **K-Means Subdivision**: Split regions with $\:{\stackrel{-}{\alpha\:}}_{k}<{\uptau\:}$​ into $\:K{\prime\:}=2$ subregions

13 $$\:\underset{\{{\boldsymbol{c}}_{1},{\boldsymbol{c}}_{2}\}}{\mathrm{min}}{\sum\:}_{i\in\:{\mathcal{R}}_{\mathcal{k}}}\underset{j=\mathrm{1,2}}{\mathrm{m}\mathrm{i}\mathrm{n}}{\Vert{\mathbf{x}}_{i}-{\boldsymbol{c}}_{j}\Vert}^{2}$$

#### Dynamic classifier ensemble

1. **Classifier Selection Rule**: For region $\:{\mathcal{R}}_{\mathcal{k}}$​, choose

14 $$\:{\mathcal{C}}_{\mathcal{k}}^{*}=\mathrm{arg}\underset{{\mathcal{C}}_{\mathcal{m}}}{\mathrm{max}}{\alpha\:}_{\mathcal{k}}^{\left(m\right)}$$

where $\:{\alpha\:}_{\mathcal{k}}^{\left(m\right)}$​ is the validation accuracy of classifier $\:{\mathcal{C}}_{\mathcal{m}}$​.

##### Theorem 2

**(Ensemble Selection Consistency)**. For region $\:{\mathcal{R}}_{\mathcal{k}}$​, let $\:{\alpha\:}_{\mathcal{k}}^{\left(m\right)}$ denote classifier $\:{\mathcal{C}}_{\mathcal{m}}$​’s validation accuracy. The optimal classifier $\:{\mathcal{C}}_{\mathcal{k}}^{*}$​ (Eq. 14) satisfies

15 $$\:\mathbb{P}\left({\alpha\:}_{\mathcal{k}}^{\left({m}^{*}\right)}\ge\:\underset{m\ne\:{m}^{*}}{\mathrm{max}}{\alpha\:}_{\mathcal{k}}^{\left(m\right)}\text{}\:\right)\ge\:1-exp\left(-2\:\left|{\mathcal{R}}_{\mathcal{k}}\right|{\left(\varDelta\:\alpha\:\right)}^{2}\right)$$

where $\:\varDelta\:\alpha\:$ is the minimum accuracy gap between $\:{\mathcal{C}}_{\mathcal{k}}^{*}$​ and suboptimal classifiers.

##### Proof

By Hoeffding’s inequality, the probability of correct classifier selection grows exponentially with region size $\:\left|{\mathcal{R}}_{\mathcal{k}}\right|$.This corollary establishes statistical guarantees for dynamic ensemble selection—a key theoretical advancement over static fusion strategies.

2. **Ensemble Prediction**:

Final label for pixel $\:{i\in\:\mathcal{R}}_{\mathcal{k}}$. During inference, ground-truth labels are unavailable. Therefore, the decision of whether to use the region-wise selected classifier must rely only on model predictions (e.g., prediction disagreement/uncertainty), rather than on $\:{y}_{{R}_{k}}$. Specifically, we define a prediction-disagreement score for each pixel based on the outputs of the classifier pool, and use it to switch between (i) a consensus prediction and (ii) the region-wise selected classifier. It should be emphasized that the proposed ensemble prediction does not always rely on the single best classifier. Instead, the ensemble mechanism is designed as an adaptive decision rule during inference. When all classifiers in the pool produce consistent predictions for a given pixel, a consensus predictions adopted to improve robustness. Conversely, when prediction disagreement is observed, indicating higher uncertainty or regional complexity, the region-wise selected best classifier is activated.

**Fig. 6:**
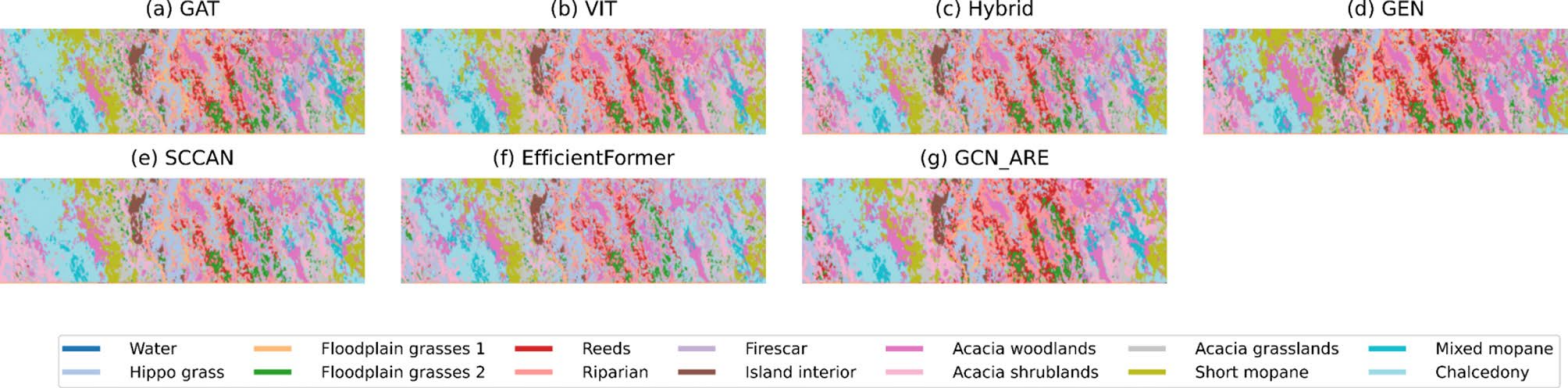
Thematic maps of Botswana.

**Table 7:** Performance of different methods versus proposed GCN-ARE for Botswana Dataset.

class	GAT^21^	ViT^25^	Hybrid^23^	GEN^19^	SCCAN^10^	EfficientFormer^26^	GCN-ARE
*1*	100.00	98.81	97.66	100.00	97.28	96.15	100.00
*2*	83.51	94.19	92.05	90.00	83.51	92.05	100.00
*3*	97.88	100.00	99.57	100.00	94.29	100.00	100.00
*4*	100.00	100.00	100.00	97.94	100.00	100.00	100.00
*5*	99.52	97.90	98.74	98.08	98.10	98.21	97.40
*6*	89.62	95.60	97.55	88.00	93.02	94.09	98.88
*7*	100.00	97.93	97.49	97.92	99.57	98.31	100.00
*8*	97.86	94.82	98.92	87.56	98.92	97.86	100.00
*9*	95.11	98.33	97.65	98.99	98.29	97.64	100.00
*10*	97.29	96.54	97.78	90.83	98.22	94.42	100.00
*11*	95.16	99.26	97.20	98.84	97.55	97.54	92.13
*12*	100.00	100.00	100.00	100.00	94.15	100.00	99.45
*13*	99.20	100.00	97.25	98.41	99.60	98.80	100.00
*14*	100.00	100.00	100.00	100.00	100.00	96.15	97.89
*OA*	96.97	98.18	98.05	96.19	97.00	97.41	**98.86**
*AA*	97.39	98.45	98.21	96.27	97.33	97.72	**98.98**
*Kappa*	0.97	0.98	0.98	0.96	0.97	0.97	**0.99**

16 $$\:{\widehat{y}}_{i}=\left\{\begin{array}{c}{\mathcal{C}}_{\mathcal{k}}^{*}\left({\mathbf{z}}_{i}\right)\mathrm{,}\text{}\text{}\text{}\text{}\text{}\text{}\text{}\mathrm{i}\mathrm{f}\text{}{\Delta}\mathrm{(}{\mathbf{z}}_{i}\text{}\mathrm{)}\mathrm{>}{\tau}\mathrm{,}\\\:{\mathcal{C}}_{\mathcal{k}}^{mv}\left({\mathbf{z}}_{i}\right),\:\:\:otherwise.\:\:\:\:\:\end{array}\right.$$

Here $\:{\mathbf{z}}_{\mathbf{i}}$denotes the embedding (node) feature of pixel $\:i$, $\:{\mathcal{C}}_{\mathcal{k}}^{*}$ is the best base classifier selected for region $\:{R}_{k}$during the validation/evaluation stage, and $\:{\mathcal{C}}_{\mathcal{k}}^{mv}$ is the majority-vote (consensus) output of the classifier pool. The threshold $\:\tau\:$controls when $\:{\mathcal{C}}_{\mathcal{k}}^{*}$ is activated under prediction disagreement.

17 $$\:\Delta\mathrm{(}{\mathbf{z}}_{i}\text{}\mathrm{)=1-}\underset{c\in\:y}{\mathrm{max}}\frac{1}{M}{\sum\:}_{m=1}1\left({\mathcal{C}}^{\left(m\right)}\left({\mathbf{z}}_{i}\right)=c\right)$$

18 $$\:{\mathcal{C}}_{\mathcal{k}}^{mv}\left({\mathbf{z}}_{i}\right)=mode\left({\left\{{\mathcal{C}}^{\left(m\right)}\left({\mathbf{z}}_{i}\right)\right\}}_{m=1}^{M}\right)$$

where $\:\mathcal{C}$ denotes the classifier pool ($\:M=3$ in our experiments). If all classifiers agree, $\:\Delta\mathrm{\:(}{\mathbf{z}}_{i}\text{}\mathrm{)}=0$; otherwise $\:\Delta\mathrm{\:(}{\mathbf{z}}_{i}\text{}\mathrm{)}>0$, indicating higher uncertainty/complexity. To avoid introducing extra hyperparameters and to improve reproducibility, we set $\:\tau\:=0$ by default, i.e., $\:{\mathcal{C}}_{\mathcal{k}}^{*}$ is activated whenever any disagreement occurs. Importantly, ground-truth labels are used only during training/validation for region evaluation and for selecting $\:{\mathcal{C}}_{\mathcal{k}}^{*}$. At inference time, Eq. (16) does not require any ground-truth information and depends solely on classifier-pool predictions through $\:\Delta\mathrm{\:(}{\mathbf{z}}_{i}\text{}\mathrm{)}$.

**Fig. 7:**
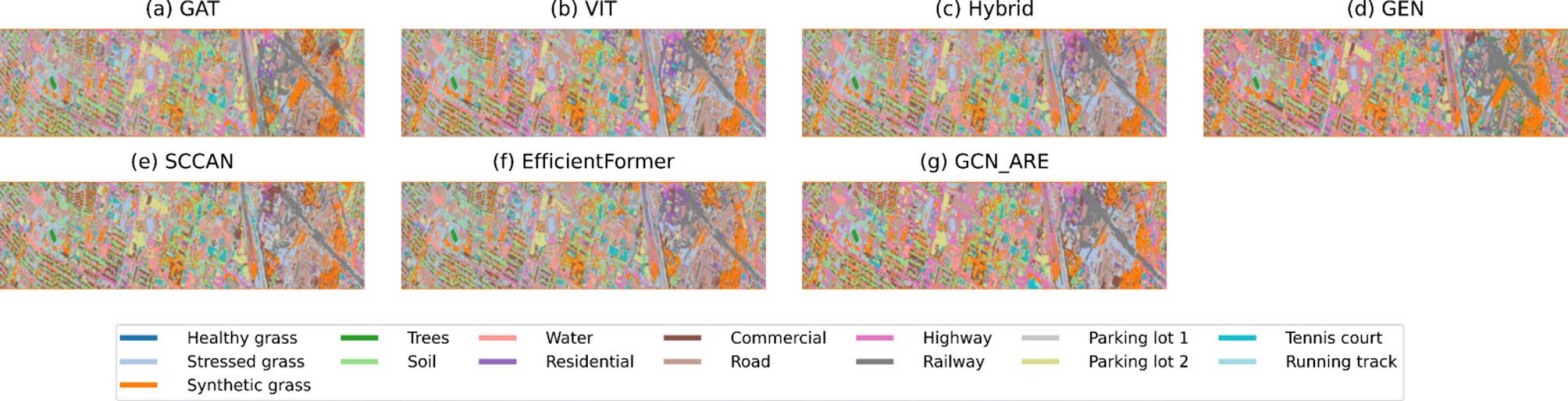
Thematic maps of Houston.

**Table 8:** Performance of different methods versus proposed GCN-ARE for Houston Dataset.

class	GAT^21^	ViT^25^	Hybrid^23^	GEN^19^	SCCAN^10^	EfficientFormer^26^	GCN-ARE
*1*	92.15	89.57	90.68	96.71	90.48	90.10	99.60
*2*	90.71	98.74	98.91	86.58	99.16	98.66	96.89
*3*	96.76	100.00	97.47	93.33	100.00	97.33	95.98
*4*	98.66	99.74	99.39	99.10	99.56	99.74	93.41
*5*	93.94	97.98	98.06	98.00	98.28	97.97	99.60
*6*	94.24	67.68	85.67	100.00	100.00	98.99	100.00
*7*	67.61	88.83	87.99	76.86	88.24	85.49	92.98
*8*	88.43	97.65	94.28	88.38	90.93	91.06	82.56
*9*	76.97	87.26	84.67	80.20	79.70	82.38	90.34
*10*	77.51	95.60	94.23	68.32	95.20	93.55	96.25
*11*	71.66	84.97	85.92	63.75	79.68	83.66	88.99
*12*	81.87	93.71	91.90	80.63	90.65	89.80	80.54
*13*	43.65	83.33	75.86	58.01	78.23	71.76	89.13
*14*	90.78	91.26	98.75	76.94	96.35	96.57	99.77
*14*	100.00	98.58	99.52	99.67	99.52	99.21	98.48
*OA*	82.57	92.53	92.39	83.21	91.52	91.35	**92.88**
*AA*	85.19	93.47	93.36	85.25	92.49	92.33	**93.63**
*Kappa*	0.81	0.92	0.92	0.82	0.91	0.91	**0.92**

3. **Majority Voting Baseline** (for comparison)

19 $$\:{\widehat{y}}_{i}=\mathrm{Mode}\left(\{{\mathcal{C}}_{\mathcal{m}}\left({\mathbf{z}}_{i}\right){\}}_{m=1}^{3}\right)$$

### Result integration

Seamlessly integrating locally optimized results into a globally consistent classification map constitutes the final challenge. This module achieves spatial coherence through hierarchical region aggregation while providing theoretical performance guarantees.

20 $$\:{\mathbb{E}}_{\left(\mathbf{x},y\right)\sim\:{\mathbb{D}}_{\mathcal{k}}}\left[L\left({\mathcal{C}}_{\mathcal{k}}^{*},x,y\right)\right]\le\:{{\epsilon}}_{\mathcal{k}}$$

where $\:{{\epsilon}}_{\mathcal{k}}$​ is the empirical risk bound for region $\:{\mathcal{R}}_{\mathcal{k}}$​.

#### Theorem 3

**(Optimal Recursive Partitioning).** Let $\:{\mathcal{R}}_{\mathcal{k}}$​ be a region with validation accuracy $\:{\stackrel{-}{\alpha\:}}_{\mathcal{k}}$​. Subdividing $\:{\mathcal{R}}_{\mathcal{k}}$​ into $\:K{\prime\:}=2$ subregions via K-means (Eq. 13) reduces the empirical risk bound $\:{{\epsilon}}_{\mathcal{k}}$​ (Eq. 19) by.

**Fig. 8:**
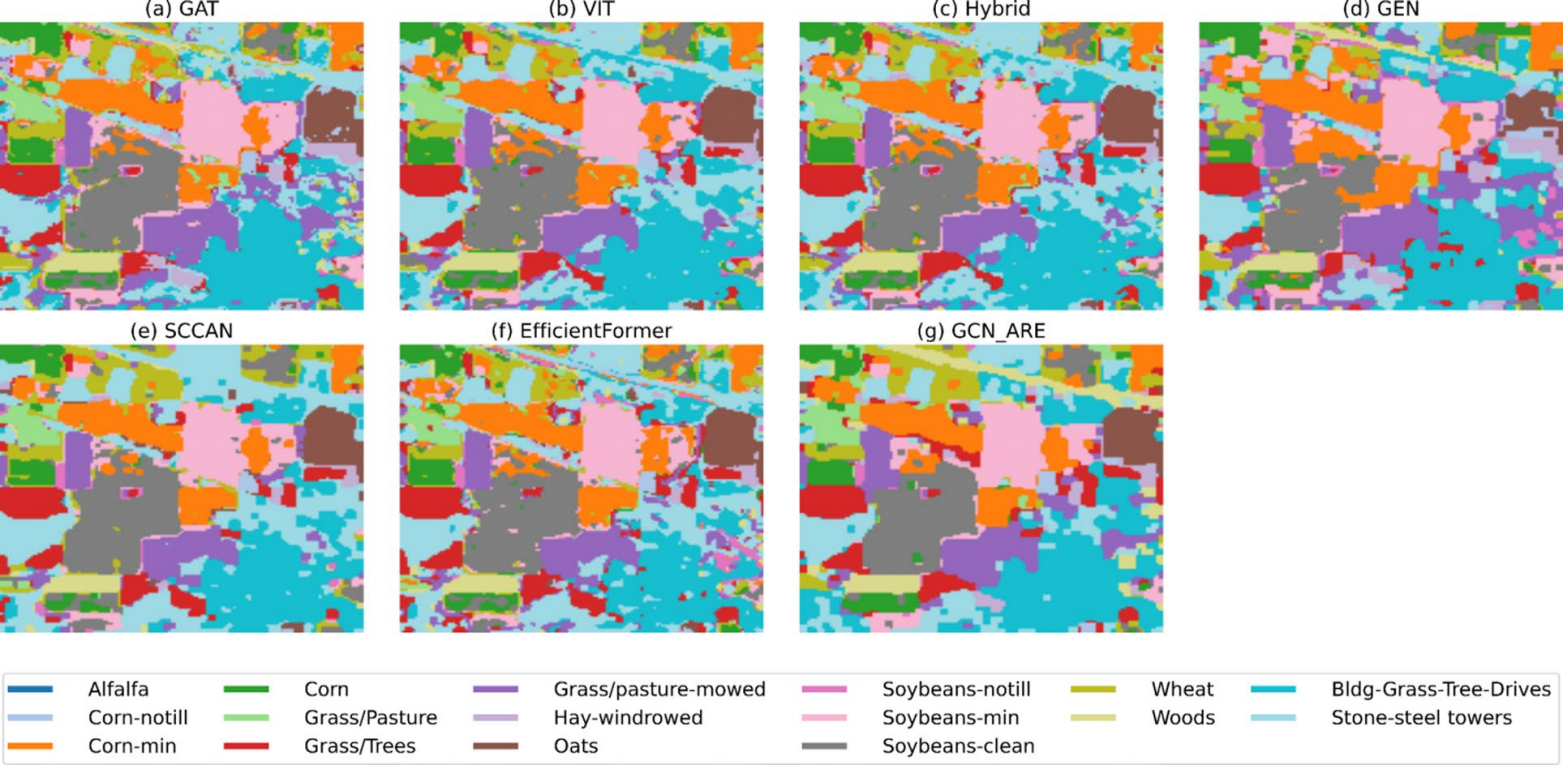
Thematic maps of IP.

**Table 9:** Performance of different methods versus proposed GCN-ARE for IP Dataset.

class	GAT^21^	ViT^25^	Hybrid^23^	GEN^19^	SCCAN^10^	EfficientFormer^26^	GCN-ARE
*1*	47.06	80.00	94.12	14.16	84.21	23.19	100.00
*2*	78.12	79.77	79.82	67.23	75.31	78.59	81.93
*3*	83.87	89.86	88.96	85.99	82.39	87.00	85.90
*4*	55.34	63.38	66.44	58.95	64.36	52.85	98.31
*5*	93.29	90.99	96.61	99.73	92.24	95.02	95.24
*6*	88.52	98.57	96.77	93.16	98.13	97.11	99.59
*7*	0.00	0.00	0.00	0.00	0.00	0.00	100.00
*8*	100.00	99.78	100.00	100.00	100.00	99.09	96.86
*9*	0.00	0.00	0.00	0.00	0.00	0.00	100.00
*10*	69.81	68.86	69.52	62.67	72.56	69.84	93.42
*11*	85.13	90.11	88.97	86.22	89.79	89.50	77.27
*12*	65.89	60.47	61.15	65.31	62.79	57.75	90.56
*13*	87.06	97.77	97.22	96.69	96.69	91.62	100.00
*14*	99.46	98.96	99.36	96.42	99.38	99.40	93.44
	68.05	76.48	69.84	82.01	73.53	76.73	98.19
	84.00	80.77	76.83	87.50	80.77	76.83	100.00
*OA*	82.04	84.30	84.07	79.30	83.76	82.68	**88.41**
*AA*	76.42	79.33	79.29	73.97	78.91	78.20	**94.42**
*Kappa*	0.80	0.82	0.82	0.76	0.82	0.80	**0.87**

21 $$\:\varDelta\:{{\epsilon}}_{\mathcal{k}}\ge\:\frac{r-{\stackrel{-}{\alpha\:}}_{\mathcal{k}}}{1+\sqrt{\left|{\mathcal{R}}_{\mathcal{k}}\right|}}$$

#### Proof

The subdivision minimizes intra-region feature variance, which directly tightens the Rademacher complexity term in Eq. 19. This result quantifies how adaptive subdivision mitigates model bias—a theoretical novelty absents in prior region-based methods.

## Results

**Fig. 9:**
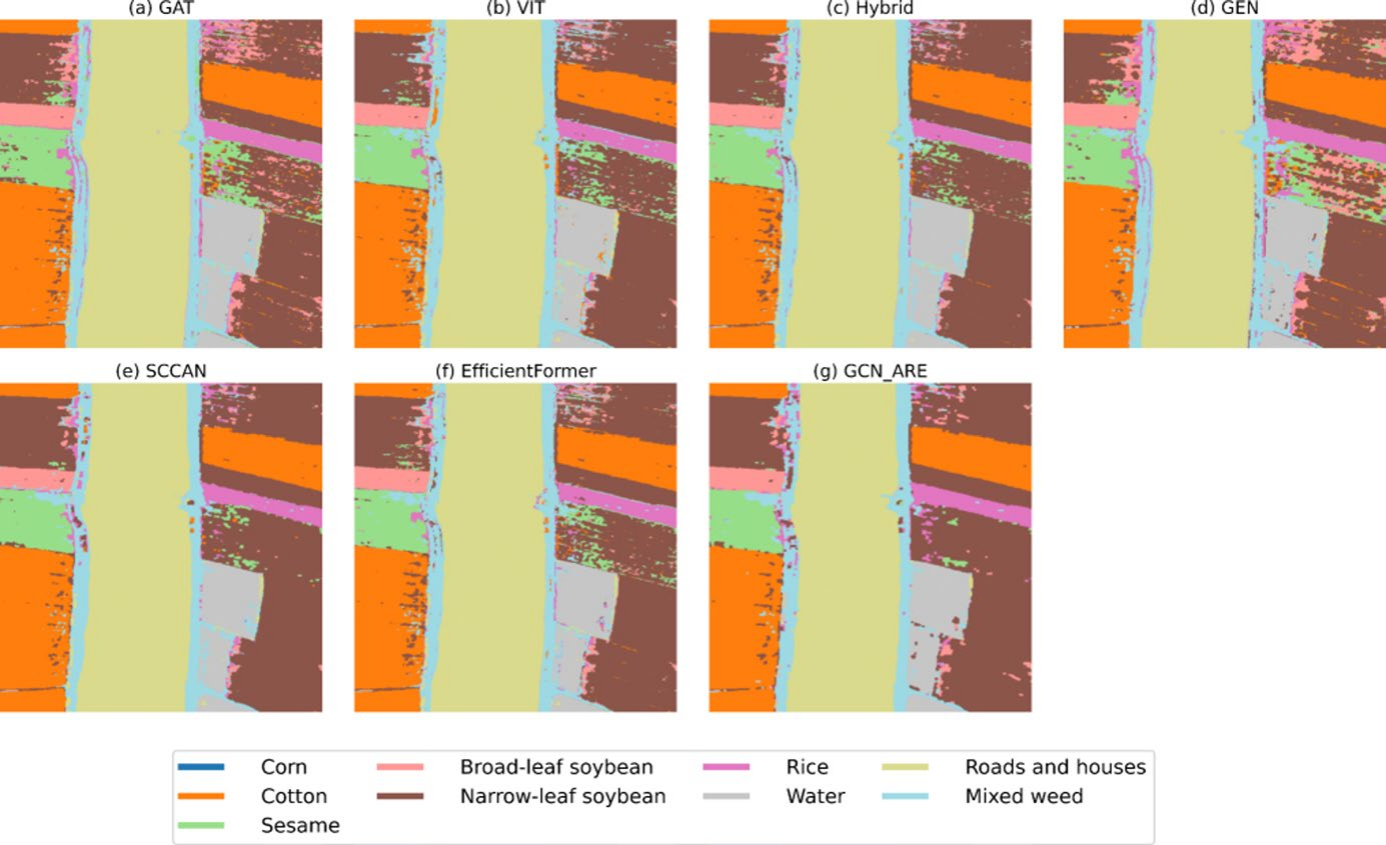
Thematic maps of LongKou.

**Table 10:** Performance of different methods versus proposed GCN-ARE for LongKou Dataset.

class	GAT^21^	ViT^25^	Hybrid^23^	GEN^19^	SCCAN^10^	EfficientFormer^26^	GCN-ARE
*1*	95.67	98.21	99.33	91.75	97.45	93.97	96.18
*2*	58.01	82.29	76.10	56.10	70.48	70.38	91.32
*3*	35.27	65.96	63.18	37.12	52.15	60.12	99.90
*4*	97.58	97.28	97.08	97.47	97.38	96.98	92.31
*5*	57.40	80.12	74.01	55.17	65.86	70.33	96.68
*6*	95.91	97.76	99.40	94.48	98.31	98.70	92.87
*7*	99.98	99.94	99.72	99.28	99.69	99.64	99.98
*8*	91.34	80.93	79.96	77.61	78.51	79.44	94.08
*9*	71.31	66.84	66.16	72.10	70.64	73.88	86.06
*OA*	90.49	94.93	94.35	89.27	93.19	93.15	**95.57**
*AA*	90.65	92.98	93.13	85.23	91.31	91.57	**94.37**
*Kappa*	0.88	0.93	0.93	0.86	0.91	0.91	**0.94**

### Data description

*1) Botswana Dataset.* Acquired through NASA’s Earth Observing-1 (EO-1) satellite mission between 2001 and 2004, this dataset covers the Okavango Delta region. The Hyperion imaging spectrometer recorded surface reflectance across 242 spectral channels (400–2500 nm spectral range) at 30 m ground resolution within a 7.7 km swath width. Post-processing removed 97 bands affected by atmospheric absorption and sensor noise, retaining 145 analyzable bands. As detailed in Table 1, the ground reference data identifies 14 distinct vegetation and land use categories. Figure 2 provides visual representations of both HSI cubes and their corresponding classification maps.

*2) Houston Dataset*. This high-resolution dataset was collected over Harris County, Texas using the ITRES CASI-1500 airborne system, featuring enhanced spatial resolution of 2.5 m. The 144-band hyperspectral imagery spans the visible to near-infrared spectrum (364–1046 nm wavelength range) with a spatial dimension of 349 × 1905 pixels. Fifteen urban and natural land cover categories are identified in the reference data, including various artificial structures and vegetation types. Comprehensive class statistics appear in Table 2, with Fig. 3 illustrating both the pseudo color composite and annotated ground truth distribution.

*3) Indian Pines Dataset.* Collected in June 1992 by the AVIRIS sensor over northwestern Indiana, USA, this benchmark dataset provides 20 m spatial resolution and 10 nm spectral resolution imagery (145 × 145 pixels, 16 land cover classes). Dominated by agricultural crops (e.g., early-growth corn/soybeans), grasslands, and woodlands, the scene exhibits distinct patchwise distributions but suffers from class imbalance due to sparse vegetation coverage (< 5%) and mixed-pixel prevalence. Class statistics are detailed in Table 3, with visualizations in Fig. 4.

*4) WHU-Hi-LongKou.* This UAV-based hyperspectral dataset was captured in July 2018 over an agricultural area in Hubei, China, using a Headwall Nano-Hyperspec sensor mounted on a DJI Matrice 600 Pro. Operating at 500 m altitude, it achieved 0.463 m spatial resolution with 550 × 400 pixels and 270 spectral bands (400–1000 nm). The scene features six crops (corn, cotton, sesame, broad/narrow-leaf soybean, rice) under clear conditions (36 °C, 65% humidity). Key parameters and class distributions are summarized in Table 4 and visualized in Fig. 5, supporting fine-scale agricultural analysis.

### Experimental setup

The proposed GCN-ARE was compared with representative state-of-the-art hyperspectral classification baselines, including Graph Attention Network (GAT)^21^, Vision Transformer (ViT)^25^, HybridSN^23^, Graph-based Ensemble Network (GEN)^19^, Spatial–Spectral Context-Aware Network (SCCAN)^10^, and EfficientFormer^26^. For the region-wise dynamic ensemble module, we employ a minimal yet representative classifier pool consisting of three lightweight base learners trained on the learned embeddings: Decision Tree (DT), SVM (RBF kernel, probability enabled), and Random Forest (RF). This design intentionally avoids relying on a large, heavily engineered pool, so that the effectiveness of our graph-based representation learning and region-adaptive dynamic selection can be validated even under a compact ensemble setting. The detailed configuration of the classifier pool is summarized in Table 5. Performance was evaluated using Overall Accuracy (OA), Average Accuracy (AA), and Cohen’s Kappa coefficient. Bold values in the tables indicate the best performance among all compared methods.

### Baseline implementations and hyperparameters

To ensure a fair and reproducible comparison, we implement all baselines using standard, publicly available modules in PyTorch and PyTorch Geometric. Specifically, GCN and GAT are instantiated via GCNConv and GATConv, respectively, while transformer-based baselines (ViT encoder, CNN–Transformer hybrid, and EfficientFormer-style lightweight transformer) are implemented using the official PyTorch TransformerEncoder building blocks. For graph-based baselines (GCN/GAT), we construct a kNN graph (k = 5, include_self = True) using scikit-learn kneighbors_graph. Unless otherwise stated, all methods are optimized with Adam (lr = 1e − 3) for 1000 epochs with cross-entropy loss on the training labels. Table 6 summarizes the key architectural hyperparameters and the unified training protocol used in our experiments.

### Classification results

The superiority of GCN-ARE is further visualized in Fig. 6, where the thematic maps exhibit consistent classification boundaries and minimal mislabeling in fragmented vegetation regions. From Table 7, the proposed GCN-ARE demonstrates superior performance on the Botswana dataset, achieving the highest OA: 98.86%, AA: 98.98%, and Kappa coefficient 0.99 among all methods. It attains perfect accuracy (100%) in 10 out of 14 classes, including critical classes such as *1*, *3*, *4*, and *7*. Notably, in class *2* and *6*, GCN-ARE outperforms all baselines by significant margins (100% vs. 83.51% for GAT in class *2*; 98.88% vs. 89.62% for GAT in class *6*). However, it slightly underperforms in class *5* (97.40% vs. GEN’s 98.08%), suggesting minor variability in challenging categories. Compared to the hybrid baseline (OA: 98.05%), GCN-ARE improves OA by 0.81%, highlighting its robustness and balanced discriminative capability across imbalanced hyperspectral data.

For the Houston dataset, GCN-ARE achieves state-of-the-art results with an OA of 92.88% in Table 8 surpassing EfficientFormer (91.35%) and ViT (92.53%). The model excels in classes 1 (99.60%), 5 (99.60%), and 14 (99.77%), demonstrating exceptional performance in critical categories. However, it shows lower accuracy in class 8 (82.56%) and 12 (80.54%), where ViT and SCCAN perform better. Despite these minor dips, GCN-ARE maintains stability, as evidenced by its highest AA and OA, outperforming the second-best baseline (ViT) by 0.35% in OA and 0.16% in AA. This underscores its generalization ability, even in datasets with complex class distributions. The effectiveness of GCN-ARE in handling extreme class imbalance is vividly illustrated in Figs. 7, 8. Table 9 dominates the IP dataset with an OA of 87.66% and an exceptionally high AA of 93.87% of GCN-ARE, outperforming all baselines by significant margins (e.g., + 3.36% OA over ViT). It achieves perfect accuracy (100%) in classes *1*, *4*, *7*, *9*, and *13*, where most baselines fail entirely (e.g., ViT and GAT score 0% in class *7*). The Kappa coefficient (0.86) further validates its reliability, surpassing the next-best method (ViT: 0.82) by 0.04. These results emphasize GCN-ARE’s capability to handle extreme class imbalances and noisy data, as seen in its dominance in low-sample classes. As shown in Fig. 7, the misclassification in class 8 (Commercial) and 12 (Parking lot2) primarily occurs in spatially adjacent regions.

**Table 11:** Runtime and memory usage comparison.

Baseline	Train time (s)	Test inference (ms/forward)	Peak CPU RSS (MB)	Peak GPU allocated (MB)	Peak GPU reserved (MB)
GCN	2.74	1.76	1169.87	58.1	1000
GAT	4.29	5.245	1215.14	201.69	1000
ViT	4.59	2.999	1237.27	86.01	1000
Hybrid	5.45	3.119	1358.98	88.18	1000
Efficient	5.74	12.062	1411.21	217.74	800
**Ours**	**1.6**	**1.682**	**1426.59**	**143.16**	**1000**

On the LongKou dataset, from Table 10 GCN-ARE achieves the highest OA (95.57%), outperforming ViT (OA: 94.93%) and Hybrid (OA: 94.35%). The thematic maps in Fig. 9 demonstrate GCN-ARE’s capability to distinguish fine-grained agricultural categories. It excels in challenging classes such as 3 (99.90% vs. ViT’s 65.96%) and 5 (96.68% vs. GAT’s 57.40%), demonstrating strong feature extraction capabilities. However, it lags slightly in class 4 (92.31% vs. GAT’s 97.58%) and 6 (92.87% vs. SCCAN’s 98.31%), suggesting room for refinement in specific spectral regions. The AA (94.37%) further highlights its balanced performance across classes, surpassing the second-best baseline (Hybrid: 93.13%) by 1.24%. These results confirm GCN-ARE’s adaptability to diverse hyperspectral landscapes and its robustness against inter-class variability.

Across all datasets, GCN-ARE consistently achieves state-of-the-art performance, with superior OA, AA, and Kappa values. Its ability to dominate imbalanced classes (e.g., IP’s class *7*) while maintaining high overall stability underscores its advanced discriminative power and generalization. Minor shortcomings in specific categories highlight opportunities for future optimization, but the method’s holistic improvements validate its effectiveness in hyperspectral classification tasks.

### Runtime and memory consumption

Table 11 reports the runtime and memory footprint of all baselines and our method on one representative dataset. Overall, our method is the most time-efficient: it yields the shortest training time (1.60 s) among all compared methods, outperforming GCN (2.74 s), GAT (4.29 s), ViT (4.59 s), Hybrid (5.45 s), and EfficientFormer (5.74 s). For test-time inference, our method also achieves the fastest per-forward latency (1.682 ms/forward), which is slightly lower than GCN (1.76 ms/forward) and substantially faster than GAT (5.245 ms/forward) and EfficientFormer (12.062 ms/forward).

In terms of memory, the peak GPU allocated memory of our method (143.16 MB) remains moderate: it is higher than lightweight baselines such as GCN (58.10 MB) and ViT (86.01 MB), but lower than GAT (201.69 MB) and EfficientFormer (217.74 MB). The peak CPU RSS shows a similar trend across methods (≈ 1.17–1.43 GB), where our method reaches 1426.59 MB. Note that “GPU reserved” mainly reflects the framework’s caching behavior and is therefore less indicative of the actual model footprint; the allocated peak provides a more faithful measure of the effective GPU memory usage. These results demonstrate that our method provides a favorable practical trade-off by delivering efficient training/inference while maintaining competitive memory consumption.

**Table 12:** Ablation Study.

GCN-ARE	Botswana	Houston	Indian Pines	WHU-Hi-LongKou
	98.86	92.88	88.41	95.57
w/o **Ada-Sd**	98.58	91.59	88.34	95.26
w/o **DES**	98.18	89.18	82.25	92.53

**Fig. 10:**
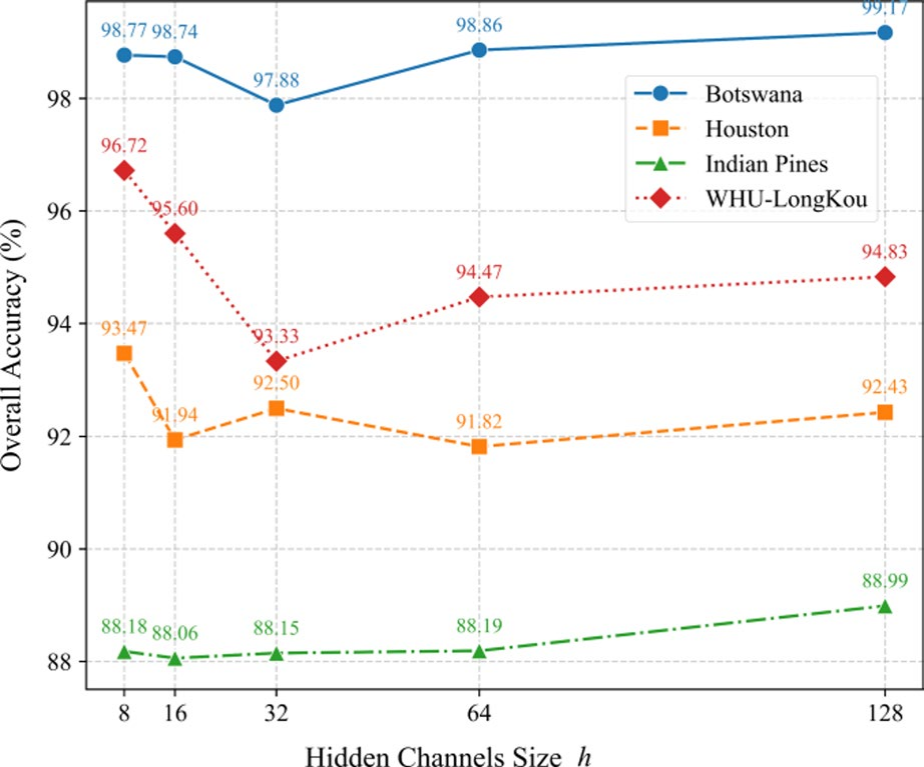
Impact of hidden channels (*h*) on OA across datasets.

**Fig. 11:**
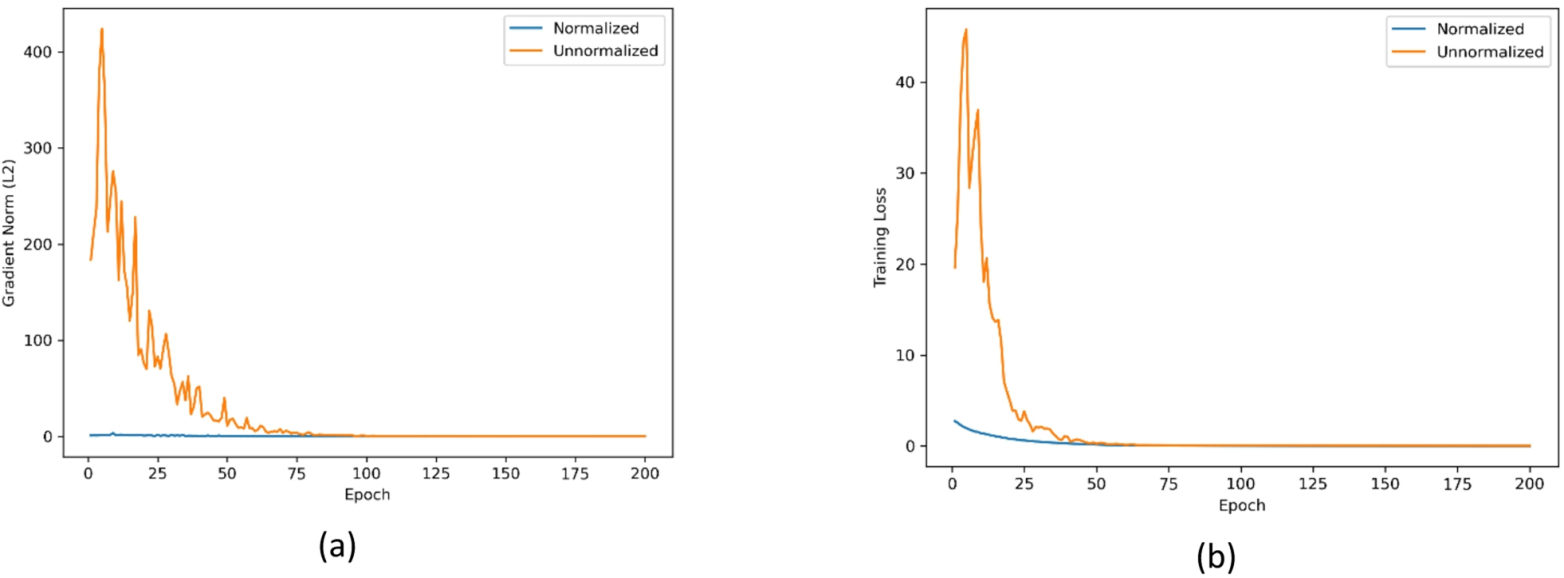
Empirical verification of Theorem 1 (spectral stability).

## Discussion

### Ablation study

From Table 12, the ablation study demonstrates the critical roles of both Ada-Sd (Adaptive Subdivision Dynamic Ensemble Selection) and DES (Dynamic Ensemble Selection) modules in enhancing the GCN-ARE framework. Removing DES caused significant performance drops across all datasets (e.g., -6.16% on Indian Pines, -3.7% on Houston), highlighting its foundational impact on classifier generalization through dynamic selection. While less pronounced, Ada-Sd removal also reduced accuracy consistently (e.g., -1.29% on Houston), suggesting its adaptive spatial partitioning strengthens local feature representation, particularly in structurally complex scenes. Notably, DES showed greater sensitivity in datasets with high inter-class spectral confusion (e.g., Indian Pines), whereas Ada-Sd proved more vital for spatially heterogeneous environments (e.g., Houston). The retained baseline performance (e.g., 92.53% on Botswana without both modules) confirms GCN-ARE’s inherent robustness, while the modular ablation validates their complementary roles in optimizing hyperspectral classification across diverse geographic scenarios.

As shown in Fig. 10, the number of hidden channels (*h*) significantly influences model performance across datasets. For Botswana, OA peaks at *h* = 128 (99.17%), while smaller values like *h* = 8 (98.77%) also perform well, suggesting moderate sensitivity to channel size. Houston achieves the highest OA (93.47%) at *h* = 8, but performance fluctuates minimally for larger h. In Indian Pines, OA gradually improves with larger *h* (88.06% at *h* = 16 vs. 88.99% at *h* = 128), indicating a slight preference for deeper architectures. Notably, WHU-LongKou performs best at *h* = 8 (96.72%), outperforming larger *h* values. This implies that smaller hidden channels may suffice for certain datasets, balancing complexity and generalization.

*Theoretical Claims Validation*.

**1. Validation of Theorem** **1****(Spectral Stability)**.

On the Botswana dataset, we conduct a controlled comparison between using a normalized Laplacian operator (“Normalized”) and an unnormalized adjacency operator (“Unnormalized”) to empirically validate the spectral-stability claim in Theorem 1. As shown in Fig. 11, the normalized operator yields a smoother and more stable optimization trajectory, where the training loss decreases steadily and the gradient L2 norm remains consistently low across epochs. In contrast, the unnormalized adjacency produces large gradient spikes and pronounced oscillations during the early training stage, accompanied by substantial loss fluctuations, indicating unstable feature propagation and gradient amplification. These observations provide empirical evidence that normalization improves training stability and supports the motivation of adopting the normalized graph operator in our framework.

**2. Validation of Theorem** **2****(Ensemble Consistency)**.

**Fig. 12:**
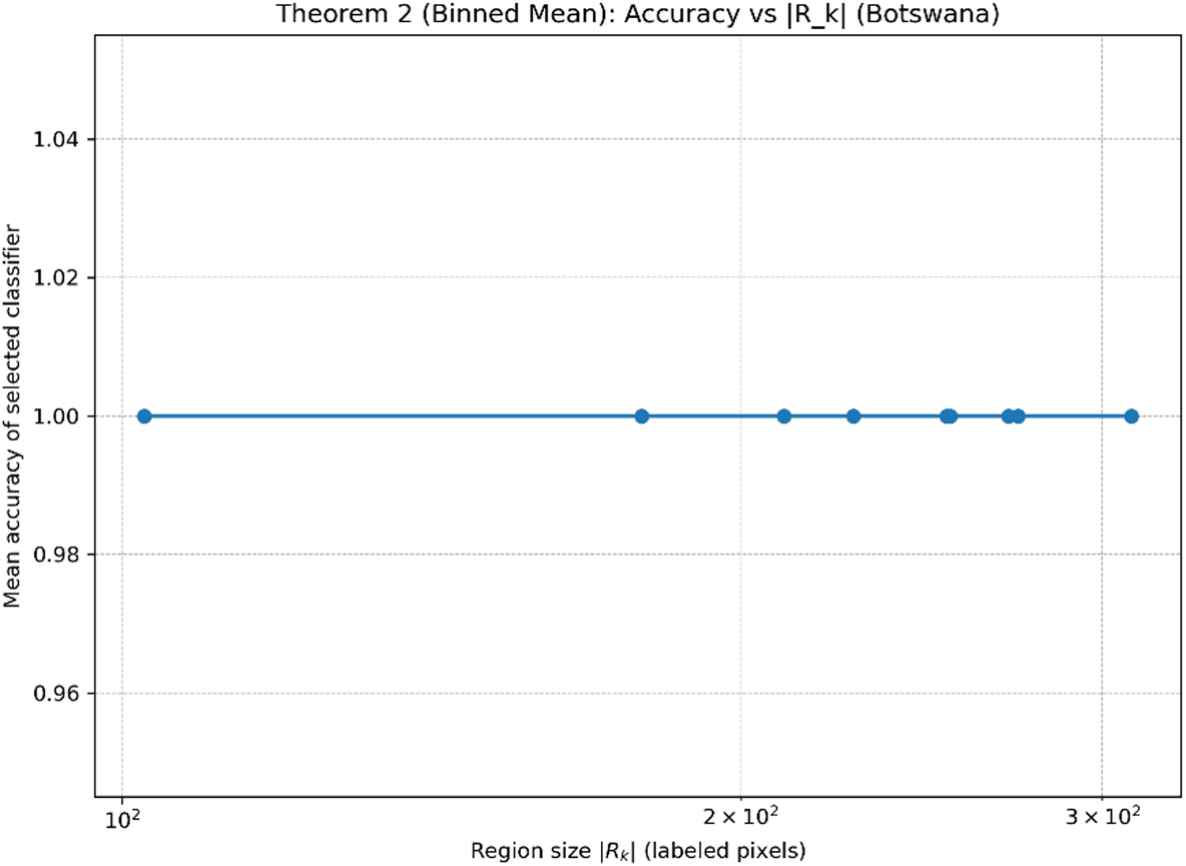
Theorem 2 Validation (Botswana): Region-level accuracy versus region size $\:\mid\:{R}_{k}\mid\:$.

**Fig. 13:**
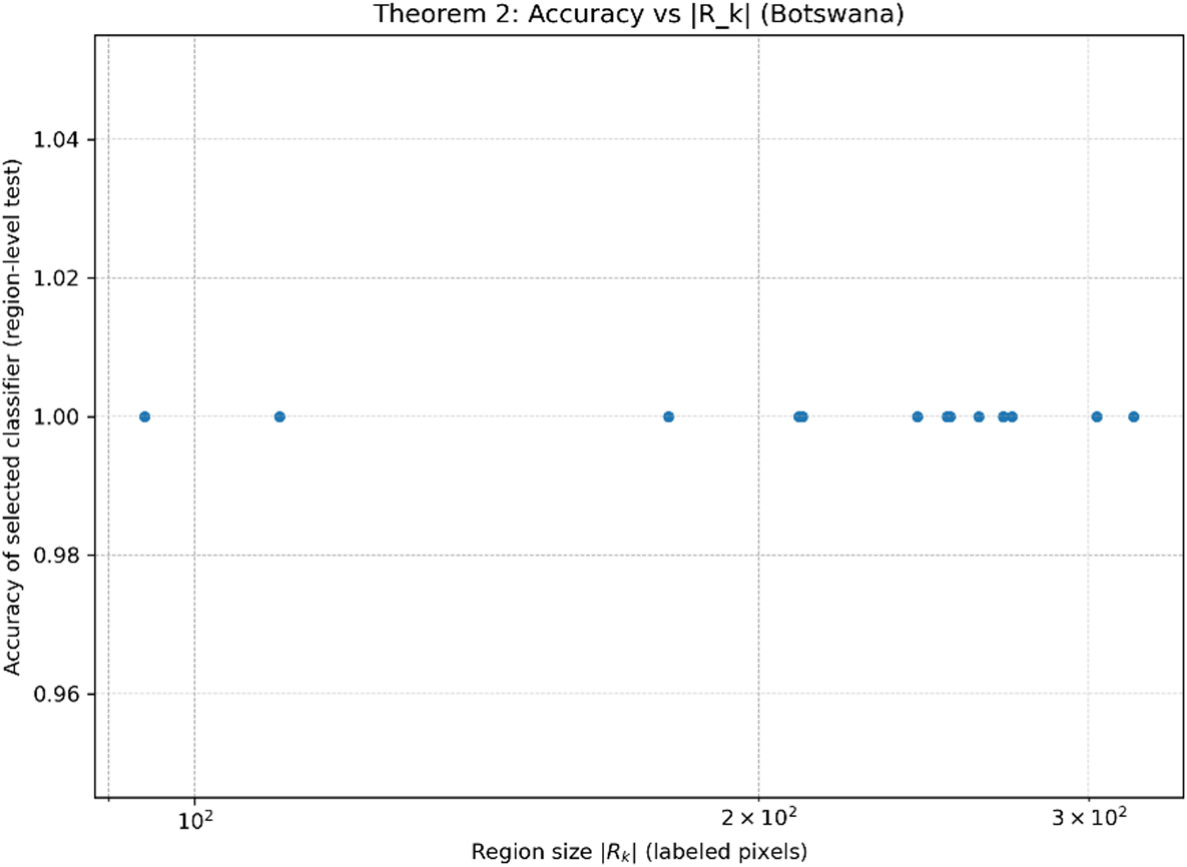
Theorem 2 Validation (Binned Mean, Botswana): Mean accuracy versus region size $\:\mid\:{R}_{k}\mid\:$. Empirical verification of Theorem 1 (spectral stability).

Figures 12 and 13 evaluate the empirical implication of Theorem 2 by examining how the region size $\:\mid\:{R}_{k}\mid\:$(measured as the number of labeled pixels contained in each refined region) relates to the region-level test accuracy of the classifier selected by the dynamic ensemble module on the Botswana dataset. In the scatter plot (Fig. 12), all regions achieve ceiling accuracy (≈ 1.0) across the observed $\:\mid\:{R}_{k}\mid\:$range (roughly $\:{10}^{2}$–$\:3\times\:{10}^{2}$ labeled pixels). The binned-mean curve (Fig. 13) remains flat at the same ceiling level, confirming that the constant trend is not an artifact of individual points.

This behavior indicates a saturation regime: the learned embeddings together with the region refinement produce locally consistent regions where the classification task becomes nearly perfectly separable, so increasing $\:\mid\:{R}_{k}\mid\:$does not further improve the observed accuracy. Importantly, this does not contradict Theorem 2; rather, it suggests that within the tested range the method already reaches the performance ceiling, making the size–generalization relationship difficult to observe numerically. Practically, these results support the claim that the proposed adaptive region mechanism yields stable and reliable region-level decisions without performance degradation for smaller regions in the evaluated scale.

3. Validation of Theorem 3 (Selection Entropy and Robustness under Embedding Perturbation).

**Fig. 14:**
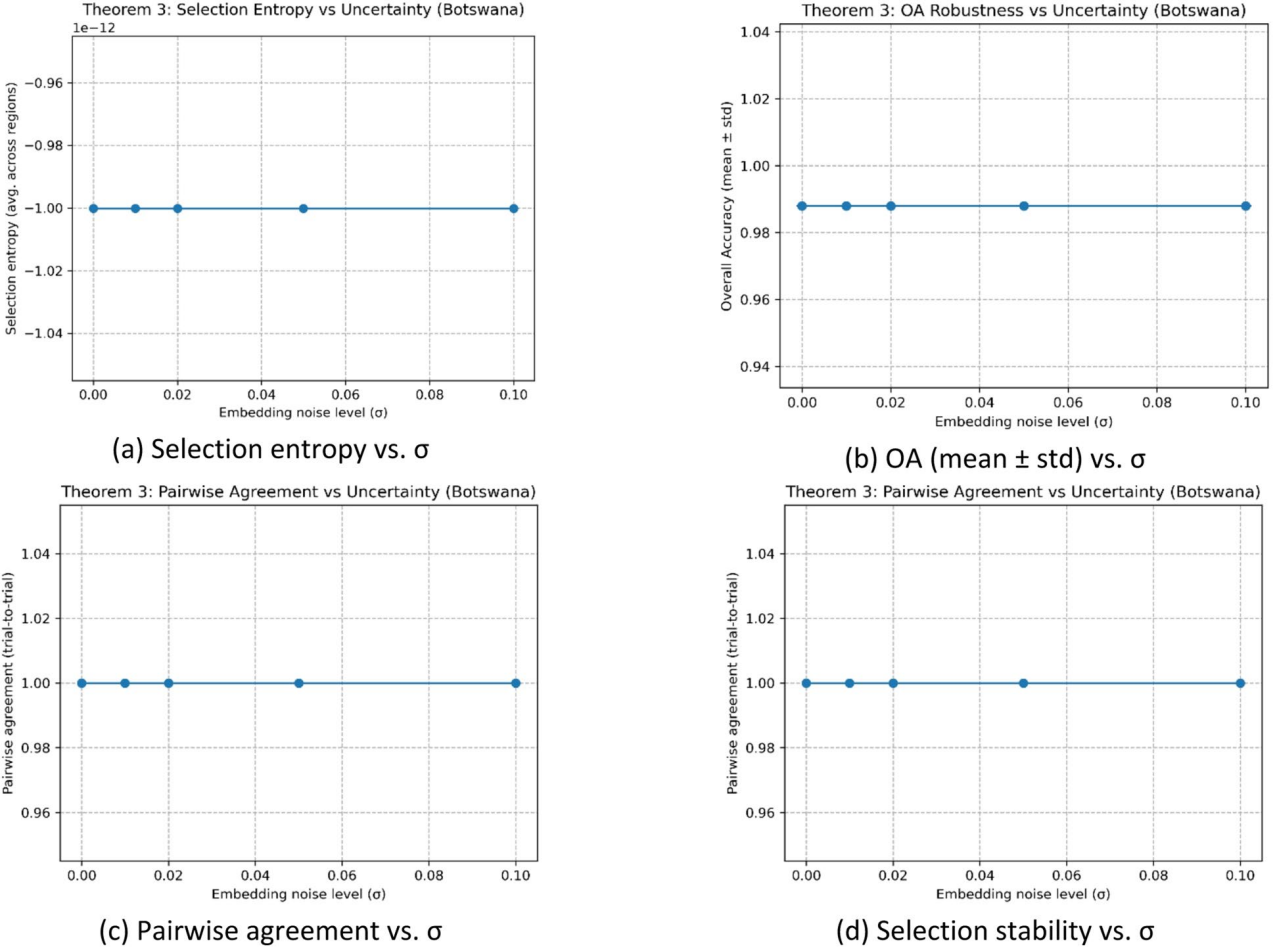
Empirical validation of Theorem 3: robustness of selection under embedding perturbation on the Botswana dataset.

Figure 14(a) reports the selection entropy as a function of the embedding perturbation level σ. The curve remains essentially unchanged across the tested noise range, indicating that the selector’s decision distribution is highly concentrated and does not become more random under moderate perturbations. Consistently, Fig. 14(b) shows that the overall accuracy (reported as mean ± standard deviation) is stable with respect to σ, suggesting that the final predictions are robust to embedding noise. At the decision-consistency level, Fig. 14(c) demonstrates near-unity pairwise agreement across trials, implying that repeated runs lead to almost identical selections/predictions rather than exhibiting stochastic fluctuations. Finally, Fig. 14(d) summarizes this behavior via the selection-stability metric (mode frequency), which also stays close to 1.0, confirming that the selected classifier (or selected configuration) is essentially invariant to σ within the evaluated range. Overall, Fig. 14(a–d) jointly support Theorem 3 by evidencing robustness of the selection mechanism and the resulting classification performance under embedding perturbations on the Botswana dataset.

### Specification of the transductive Semi-Supervised protocol

To avoid ambiguity, we explicitly specify that the “semi-supervised” setting in this work follows a transductive protocol. The graph is constructed over the entire non-background scene, and message passing is performed on all nodes. However, the optimization uses cross-entropy supervision only on a small labeled training subset (20 labeled pixels per class), while the remaining nodes—including labeled pixels reserved for validation/testing—participate in representation learning only through graph propagation. The resulting labeled/unlabeled composition and split statistics are summarized in Table 13, with per-class counts provided in Table 14.

**Table 13:** Transductive semi-supervised protocol and labeled/unlabeled composition (Botswana).

Dataset	Graph scope	Labeled per class (Train / Val)	Total labeled (Train / Val / Test)	Unlabeled nodes used in propagation	Training objective
Botswana	Full non-background scene	20 / 10	280 / 140 / 2828	377,576	CE loss on train labels only; message passing on full graph

**Table 14:** Per-class labeled split statistics under the transductive semi-supervised protocol (Botswana).

Class (gt)	Train	Val	Test	Total
1	20	10	240	270
2	20	10	71	101
3	20	10	221	251
4	20	10	185	215
5	20	10	239	269
6	20	10	239	269
7	20	10	229	259
8	20	10	173	203
9	20	10	284	314
10	20	10	218	248
11	20	10	275	305
12	20	10	151	181
13	20	10	238	268
14	20	10	65	95

### Parameter sensitivity analysis

#### Impact of hidden channels h on model performance

**Fig. 15:**
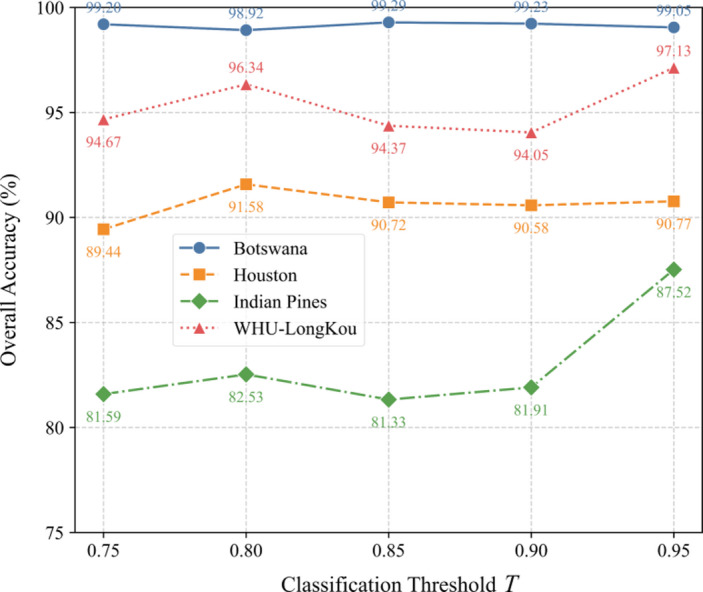
Effect of subdivision threshold (T) on OA.

**Fig. 16:**
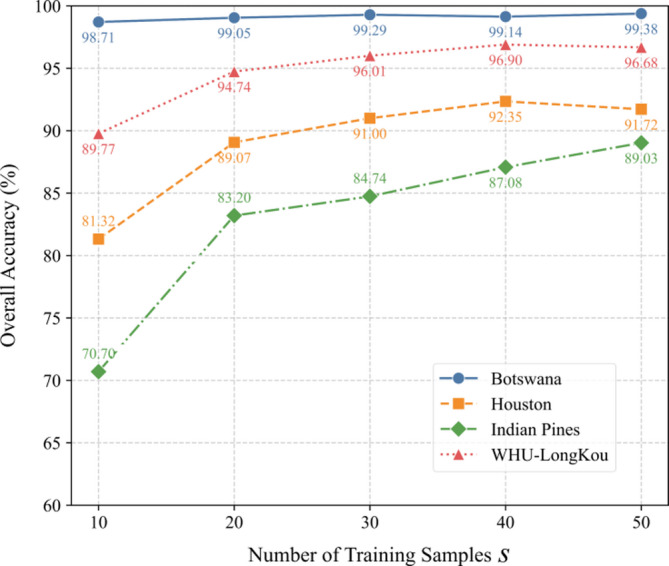
Training sample size (*S*) vs. OA.

### Impact of subdivision threshold T on model performance

Figure 15 reveal nuanced effects of *T*. For Botswana, OA remains stable (~ 99.2%) across most *T* values, peaking slightly at *T = 0.85* (99.29%). Houston shows optimal performance at *T = 0.80* (91.58%), with a notable drop at *T = 0.75* (89.44%). Surprisingly, Indian Pines achieves its highest OA (87.52%) at *T = 0.95*, suggesting stricter thresholds improve accuracy for complex classes. In WHU-LongKou, OA peaks at *T = 0.95* (97.13%), indicating that higher thresholds enhance decision boundaries in this dataset. Overall, *T* requires careful tuning, as overly strict or lenient thresholds may degrade performance depending on data characteristics.

### Impact of training sample size S on model performance

**Table 15:** Complexity analysis of key Modules.

Module	Time Complexity	Space Complexity
Graph Construction	$\:O\left(N\right)$	$\:O\left(N\right)$
GCN Training	$\:O(T\times\:NCH)$	$\:O(T\times\:NCH)$
Region Evaluation	$\:O(R\times\:{S}^{3})$	$\:O(NH+CH+{H}^{2}+HK)$
Adaptive Subdivision	$\:O(R{\prime\:}\times\:SKI)$	$\:O\left(R{\prime\:}S\right)$
Dynamic Ensemble Selection	$\:O(R\times\:M)$	$\:O\left(R\right)$
Result Integration	$\:O(N\times\:HW)$	$\:O\left(HW\right)$

*Notations*: $\:N$: valid pixels;$\:\:C,H,K$: input/hidden/class dimensions;$\:\:R,R{\prime\:}$: total/low-performance regions; $\:S$: average region size; $\:M$: classifier count; $\:I$: K-means iterations.

From Fig. 16, increasing *S* generally improves OA but with diminishing returns. For Botswana, OA stabilizes near 99.3% at *S = 30–50*. Houston peaks at *S = 40* (92.35%), then slightly declines at *S = 50* (91.72%), hinting at potential overfitting. Indian Pines exhibits steady improvement (70.70% at *S = 10* vs. 89.03% at *S = 50*), emphasizing the need for sufficient samples in complex tasks. WHU-LongKou shows consistent gains, reaching 96.90% at *S = 40*. These results underscore the importance of balancing sample size with computational costs, as marginal gains diminish beyond a critical point.

### Algorithm complexity analysis

The computational complexity of the proposed framework is analyzed in terms of both time and space complexity, as summarized in Table 15. The primary time-consuming components lie in the GCN training and region evaluation stages. Specifically, the GCN’s forward-backward propagation scales linearly with the number of nodes $\:N$ and quadratically with the hidden dimension $\:H$, leading to$\:\:O(T\times\:NCH)$ complexity for $\:T$training epochs. For region evaluation, the cubic complexity $\:O\left({S}^{3}\right)$ of training classifiers (e.g., SVM) on large regions with $\:S$ samples may become prohibitive. Spatially, the graph adjacency matrix and intermediate GCN features dominate memory usage, requiring $\:O\left(N\right)$ and $\:O\left(NH\right)$ storage, respectively.

### Limitations of proposed algorithm

While the proposed GCN-ARE framework achieves state-of-the-art performance across multiple HSI datasets, it exhibits several limitations. First, the recursive K-means-based region subdivision is sensitive to initial parameterization, potentially propagating errors in complex terrains. Second, the cubic computational complexity of region evaluation and quadratic memory overhead for graph structures hinder scalability to large-scale hyperspectral imagery. Third, despite overall robustness, performance gaps persist in specific classes (e.g., class 3 and 11 in Indian Pines), suggesting limitations in capturing nuanced spectral-spatial patterns. Additionally, sensor noise or residual artifacts may destabilize graph construction and feature propagation. Future work should address these challenges through lightweight approximations and noise-resilient modeling.

### Future work

When extending GCN-ARE to million-pixel hyperspectral scenes, the main bottlenecks are graph construction and multi-layer message passing. For a sparse graph with $\:N$nodes and $\:E$edges, the computational and memory costs roughly scale with $\:\mathcal{O}(L\cdot\:E\cdot\:F)$and $\:\mathcal{O}(N\cdot\:F+E)$, respectively, making pixel-level full-scene transductive propagation potentially expensive at $\:N\approx\:{\hspace{0.17em}}{10}^{6}$^27^. To improve scalability, we plan to explore lightweight approximations: (i) efficient propagation via lightweight filtering (e.g., ARMA-style) to reduce computational overhead^28^; (ii) region-level graph coarsening by aggregating pixels into homogeneous regions (superpixels) to reduce node/edge counts^29^; (iii) adaptive sparse topology learning and edge sparsification to keep only informative connections^30^; and (iv) lightweight modules for robust representation under sparsity, such as adaptive-filter fusion, multi-branch collaboration, and attention/adaptive receptive-field aggregation^31–34^. These directions will enable scalable GCN-ARE variants for very large scenes while maintaining a practical accuracy–efficiency trade-off.

## Data Availability

The datasets analyzed in this study are publicly available: the **Botswana** dataset from NASA’s EO-1 Hyperion mission (IEEE GRSS Data Fusion Contest), the **Houston** dataset from the IEEE GRSS 2013 Data Fusion Contest, the **Indian Pines** dataset from Purdue University’s MultiSpec, and the **WHU-Hi-LongKou** dataset released by Wuhan University. All datasets can be freely accessed for research purposes. Processed data and codes supporting the findings of this study are available from the corresponding author upon reasonable request.
